# Procedural analgesia with nitrous oxide at home for epidermolysis bullosa: A case report

**DOI:** 10.1097/MD.0000000000028474

**Published:** 2022-01-07

**Authors:** Manuel Murciano, Claudia Laterza, Ettore Attolini, Sonia Storelli, Giovanni Dipietro, Antonio Rubino, Giuseppina Annicchiarico

**Affiliations:** a Regional Coordination of Rare Diseases (CoReMaR), Apulia Regional Agency for Health and Social Care (AReSS), Bari, Italy; b Emergency and General Pediatrics Department, Bambino Gesù Children's Hospital, IRCCS, Rome, Italy; c Health System Innovation and Quality Area at Apulia Regional Agency for Health and Social Care (AReSS), Bari, Italy; d Department of Anesthesia and Resuscitation, Valle D’Itria Hospital Martina Franca, Taranto, Italy.

**Keywords:** epidermolysis bullosa, nitrous oxide, procedural pain, rare disease, wound care

## Abstract

**Rationale:**

Epidermolysis bullosa (EB) is an inherited disease characterized by fragile skin with painful blistering, which requires lifelong skin and wound care. This case report describes the use of inhaled nitrous oxide (N_2_O) for procedural pain control at home during wound care in a young man with severe dystrophic EB. To our knowledge, only 1 case was reported by Ingelmo et al in 2017 regarding the use of N_2_O at home in a 4-year-old-child. To date, no such attempt has been made in adult patients.

**Patient concerns:**

Our patient was a 28-year-old man. Frequent blisters appear spontaneously, and are often preceded by erythema and itching. Patient required daily treatment daily consisting of lancing blisters with a needle and emptying them by compression.

**Diagnoses:**

Severe recessive dystrophic EB diagnosed at the time of delivery.

**Interventions:**

Procedural pain control was managed by the auto-administration of an inhaled N_2_O and air gas mixture.

**Outcomes:**

Conscious sedation with N_2_O leads to beneficial effects, such as reduction in dressing duration, acute procedural pain, local antibiotic needing, medication memory, anxiety, anticipatory pain, and fatigue after the dressing session.

**Lessons:**

N_2_O analgesia is safe and effective, resulting in a significant reduction in procedural pain and an improvement in the quality of life of patients and their caregivers.

## 1 Introduction

Epidermolysis bullosa (EB) is a rare disorder mainly characterized by skin diseases and multi-system effects. Four major types of EB are known ^[1]^ and are categorized as Simplex, Junctional, Dystrophic (recessive or dominant) and Kindler Syndrome; there are at least 18 genes associated with the different types of EB.^[1]^ Patients present acute, chronic and procedural pain care needs ^[2]^ depending on EB types. Skin pain represents a routine for many patients with EB and is related to sites of blistering, skin loss, and infection, with potential development of chronic and neuropathic pain.^[3]^ While not pain per se, itching is a major source of discomfort ^[4]^ that affects the quality of life ^[5,6]^ and drug treatments can further impair activities of daily living.^[7]^ Current practices for pain management emphasize psychological interventions, pharmacological therapies, and local wound care.^[7]^ It is important to discuss the needs of the patient and the options for caring for those needs with the patient and family to draw up an individualized care plan. Owing to the low prevalence of EB, expertise in pain care is often restricted to a few specialized care centers. Furthermore, evidence-based pain care is limited by the near absence of scientific literature specific to EBs. Inhaled nitrous oxide has been suggested for wound care in EB, in some texts and review articles,^[8,9]^ but its use at home in EB patients has been poorly studied. This case report is based on the pain care guidelines requested by the Dystrophic Epidermolysis Bullosa Research Association International ^[7]^ and a single case report of a 4-years-old child in 2017.^[10]^

## 2 Case presentation

Our patient was a 28-year-old man with severe recessive dystrophic EB diagnosed at the time of delivery. Frequent blisters appear spontaneously, and are often preceded by erythema and itching. The patient's medical history revealed episodic bone microfractures due to severe osteoporosis (despite vitamin D supplementation), celiac disease, and an episode of severe post infectious hemorrhagic vasculitis. Multiple surgeries have been performed for scarring pseudosyndactyly to improve hand deformities and the inability to perform fine manipulations. Chronic therapy includes betamethasone, ebastine, and oxycodone/naloxone (40/20 mg/day) for basal pain control, administered after other alternatives have proven to be inadequate. Acetaminophen, nonsteroidal anti-inflammatory drugs, and morphine do not provide the desired analgesia without long-lasting side effects (such as sedation and constipation). Denosumab was administered monthly to treat the osteoporosis.

Blister treatments are also essential, as lesions will extend rapidly if undrained. This treatment is daily and consists of lancing blisters with a needle and emptying them by compression. Furthermore, this patient requires an average of 3 full-body dressings per week, and wound care is very painful, especially on the feet and armpits. He complained of intense burning pain during removal and wound cleansing, and the pain was often so intense that his caregivers could not conduct them adequately. He presented with hyperalgesia, allodynia, anticipatory pain reaction, and anxiety just before the procedure. Topical treatments were ineffective in controlling the procedural pain. Among the short-acting opioids, only immediate-release oral morphine is allowed in Italy for non-oncological pain, but the patient feels sleepy, drowsy, or lightheaded and tolerates it poorly.

He requested a short-acting, effective analgesic strategy, with no residual effects and minimal long-term consequences.

The initiation of the procedure was decided by pain therapy experts regardless of data collection.

According to the patient and his family, we started administering nitrous oxide (N_2_O) through a nasal mask for procedural pain management during advanced dressing.

In fact, the key features of nitrous oxide include reliable analgesia with rapid onset and offset and the possibility of patient self-regulation, as well as effective pain relief and a sedative effect without loss of consciousness, reducing patient anxiety with minimal side effects. Before the first procedure, we evaluated the patient (including pain expert assessment), asked for his or her issues and expectations, obtained informed consent from the patient, the willingness of the family to cooperate, and authorization by the local health authority. Due to the inactivation and depletion of vitamin B12 by N_2_O, its long-term use has been associated with myelopathy, manifested clinically by paraparesis or impaired sensation, and megaloblastic anemia.^[10–12]^ The risk is increased in patients suffering from malnutrition, which is common among EB patients, so we planned a basal control (vitamin B12, serum folate levels and red and white blood cell counts are normal) and periodic follow up (every 2 months) to assess neurologic symptoms and monitoring vitamin B12 levels and blood counts.^[13]^ During the first administration an experienced anesthesiologist was present. For the following, we only required the presence of a doctor with airway management skills and basic life-support certification. A room in the patient's home was set aside for the procedure, professionally cleaned, aerated, and equipped with all emergency devices. Continuous recording of vital signs, including oxygen saturation, was provided using a multi-parameter monitor. The home delivery system consisted of an IntelliFlux (Master Flux Plus, Tecno-Gaz, Italy) equipped with a gas mixer (N_2_O and O_2_). It includes a pressure-reducing regulator, a backflow one-way demand valve, and a nasal mask, permitting the flow of N_2_O during inspiration only. Exit gas with non-return valves reduces N_2_O emissions in the environment. The nose mask had a perfect hold. The adaptive cushioning and front forks enable perfect, stable positioning, thus preventing any dispersion or leakage of gas, and the central connection contains non-return valves. The “Y” Connection allows the proper flow of gas mixture preventing that the exhaled gases can return to power supply circuit. The small chamber eliminates the risk of CO_2_ permanence. The first home administration for conscious sedation was conducted in December 2020. Advanced dressings have a frequency of 3 times per week. The most painful phase is the removal of old dressings and wound cleansing. The gas concentration was set to a N_2_O:O_2_ ratio of 30:70 (less than the 50:50 fixed mixture present in the pre-filled cylinders). Conscious sedation and cough reflex are preserved, but a fasting period (2 hours) is observed before the procedure, as a safety precaution, to avoid any risk of vomiting and inhalation, although. During the home sessions, the patient is cooperative, and he gives signals within 2 to 3 minutes. The advanced dressings carried out in a single session that involved half of the body, alternatively the upper body, armpits (45 minutes required), and lower body (30 minutes required). Dressings were performed 48 hours apart. The beneficial effects of conscious sedation are evident. First, dressings were easily completed in a significantly reduced time of 25% to 40% from a duration of 3.5 to 4 hours to a duration–1 to 15 hours. Second, many benefits for the patient have been highlighted: he reports a significant acute-pain reduction, reduction in anticipatory pain and anxiety before the procedure, less medication memory, and less fatigue after the dressing session, which allows him to enjoy and perform multiple activities during the day. The domestic device allows greater flexibility of schedules, allowing less effort for caregivers; therefore, their burden has been significantly reduced in parallel with the reduction in patient suffering. The medium-term benefits are a marked improvement in the bad smell of wounds up to the disappearance of odors and the reduction of the tubes of antibiotic cream applied for local infection (from 1 per month to half a tube per month). His saturation and vital signs remained stable, and there were no episodes of desaturation, tachycardia, or hypotension. We measured pain using the visual analog scale (VAS) with and without conscious sedation with values of 100 mm (10 cm) and 10 mm (1 cm), respectively. In addition, the Numeric Pain Rating Scale (NPRS) values decreased from 10 to 1 during dressing with N_2_O oxide administration. The patient was scheduled to undergo regular follow-up by a pain specialist. The patient received a standard multivitamin supplement daily. He now performs regular daily social activities.

## 3 Discussion

Nitrous oxide has been used for painful procedures both in and out of hospital settings,^[14]^ including dental clinics and emergency departments.^[15]^ It is available in both fixed 50:50 and variable combinations with oxygen. In Italy, nitrous oxide in extra-hospital settings can be used in cylinders in a mixture of 50% with oxygen weight equal to or less than 20 kg. For the exclusive use of specialists in anesthesia and resuscitation and dentistry.^[16]^ Regarding adverse effects, N_2_O may cause nausea, vomiting, headache, and dizziness, with severe acute complications being very rare.^[15]^ Depending on the delivery system (mouth piece, face mask, or nasal mask) concerns arise about environmental pollution and exposure of health care personnel.^[14]^ Epidemiological studies have linked long-term occupational exposure to N_2_O with spontaneous abortion, congenital anomalies, and reduced rates of fertility.^[17]^ Despite safety features, a certain degree of contamination will be produced by N_2_O exhaled from the patient. The National Institute for Occupational Safety and Health recommended exposure limit is 25 parts per million (p.p.m.) as a time-weighted average in the operating room during the period of general anesthesia.^[18]^ The N_2_O concentration after 15 minutes of auto administration by a patient with an on-demand valve system in a 10 m^2^ emergency room without evacuation system has been estimated to be of 4.2 time-weighted average- 8 hours p.p.m.^[10]^ This scenario resembles the conditions of our clinical case, which presents 2 to 3 times of the N_2_O exposure (30–45 minutes maximum) but a longer washout time (48 hours compared with 20 minutes in the clinical simulation). The administration of an N_2_O/O_2_ mixture in well-ventilated rooms with an on-demand valve and for short-duration procedures would ensure that N_2_O exposure remains below the critical values.^[18]^ An alternative option may be to use a delivery system incorporating a double-face mask and a portable scavenging device, the use of which has been shown to maintain ambient N_2_O levels within the recommended limits.^[19]^

In our experience, N_2_O analgesia has been shown to be safe and effective, resulting in a significant reduction in procedural pain and an improvement in the quality of life of patients and their caregivers. However, the home use of this method cannot be generalized to all patients with EB because, for example, some patients may not be able to tolerate the skin rubbing effect of the face mask, although the nasal mask may be a better tolerated alternative because of its lower skin interface, which reduces friction. The potential for pulmonary aspiration in non-fasted patients is an additional concern associated with home N_2_O. In our patient, the use of this agent was limited to a self-administered basis, and titration was performed to achieve analgesia without significant sedation or loss of protective airway reflexes. Esophageal strictures, which were not present in our patient, may be a concern for increased aspiration risk in other EB patients.^[10]^ Moreover, in our case, the patient was an adult and therefore able to provide cooperation and competent feedback, unlike young children with severe forms of EB who need pain-free procedures to reduce psychosocial burden. Finally, the home management of nitrous oxide requires a team of healthcare professionals and assistants capable of providing adequate monitoring during the procedure (including oxygen saturation and measurement of vital signs) and careful follow-up and management of potential side effects. We advocate home availability of both nitrous oxide delivery devices and a team of experts. Obviously, this home-based intervention offered by the public health system cannot, by any means, be considered entirely conclusive and requires further experience to improve the procedures and ensure patient safety. However, the introduction of a public health team into a home setting for procedural pain control in these patients resulted in clear benefits to nursing home operations and patient care facilities.

The staff became aware of the patients’ physical, social, and psychological needs, and the patients naturally benefited from these changes in the medical care and pain control programs introduced into the home. The authors believe that further studies are needed to implement this method of home procedural analgesia for pain control, as provided by Italian Law 38/2010.^[20]^

## Acknowledgments

The authors acknowledge Giovanni Gorgoni, Chief Executive Officer of the Apulia Regional Agency for Health and Social Care (AReSS) for his teachings in strategic planning of health and social policies. Giovanni Gorgoni provided permission to be named.

## Author contributions

**Conceptualization:** Manuel Murciano, Claudia Laterza, Ettore Attolini, Sonia Storelli, Giovanni Dipietro, Antonio Rubino, Giuseppina Annicchiarico.

**Methodology:** Manuel Murciano, Antonio Rubino, Giuseppina Annicchiarico.

**Project administration:** Ettore Attolini, Giuseppina Annicchiarico.

**Supervision:** Manuel Murciano, Claudia Laterza, Ettore Attolini, Sonia Storelli, Giovanni Dipietro, Antonio Rubino, Giuseppina Annicchiarico.

**Validation:** Manuel Murciano, Claudia Laterza, Ettore Attolini, Sonia Storelli, Giovanni Dipietro, Antonio Rubino, Giuseppina Annicchiarico.

**Visualization:** Antonio Rubino.

**Writing – original draft:** Manuel Murciano, Claudia Laterza.

**Writing – review & editing:** Claudia Laterza.
